# Supplementary material for: Eicosanoid and eicosanoid-related inflammatory mediators and exercise intolerance in heart failure with preserved ejection fraction
Source: Nat Commun. 2023 Nov 20;14:7557. doi: 10.1038/s41467-023-43363-3 (PMC10662264; doi:10.1038/s41467-023-43363-3)

Eicosanoid and Eicosanoid-Related Inflammatory Mediators and Exercise Intolerance in Heart Failure with Preserved Ejection Fraction

Supplemental Appendix

Supplemental Appendix Table of Contents

Supplementary Figure 1. Heatmap for associations of the 70 HFpEF-related eicosanoid and eicosanoid-related metabolites with exercise traits in the MGH CPET Sample (alternate color scheme).

Supplementary Figure 2. Principal component score for principal components 1 and 2 for exercise traits.

Supplemental Figure 1. Heatmap for associations of the 70 HFpEF-related eicosanoid and eicosanoid-related metabolites with exercise traits in the MGH CPET Sample (alternate color scheme). Color coding represents standardized ß-coefficient in primary model (X-SD change in exercise trait per 1-SD change in eicosanoid metabolite). Primary analyses are adjusted for age, sex, and plate number. Clustering is based on exercise traits (columns). Color scale indicates positive associations in blue, and negative associations in yellow. Abbreviations: CO = cardiac output, HR = heart rate, PAP = pulmonary artery pressure, PCWP = pulmonary capillary wedge pressure, SBP = systolic blood pressure. Source data are provided as a Source Data file.

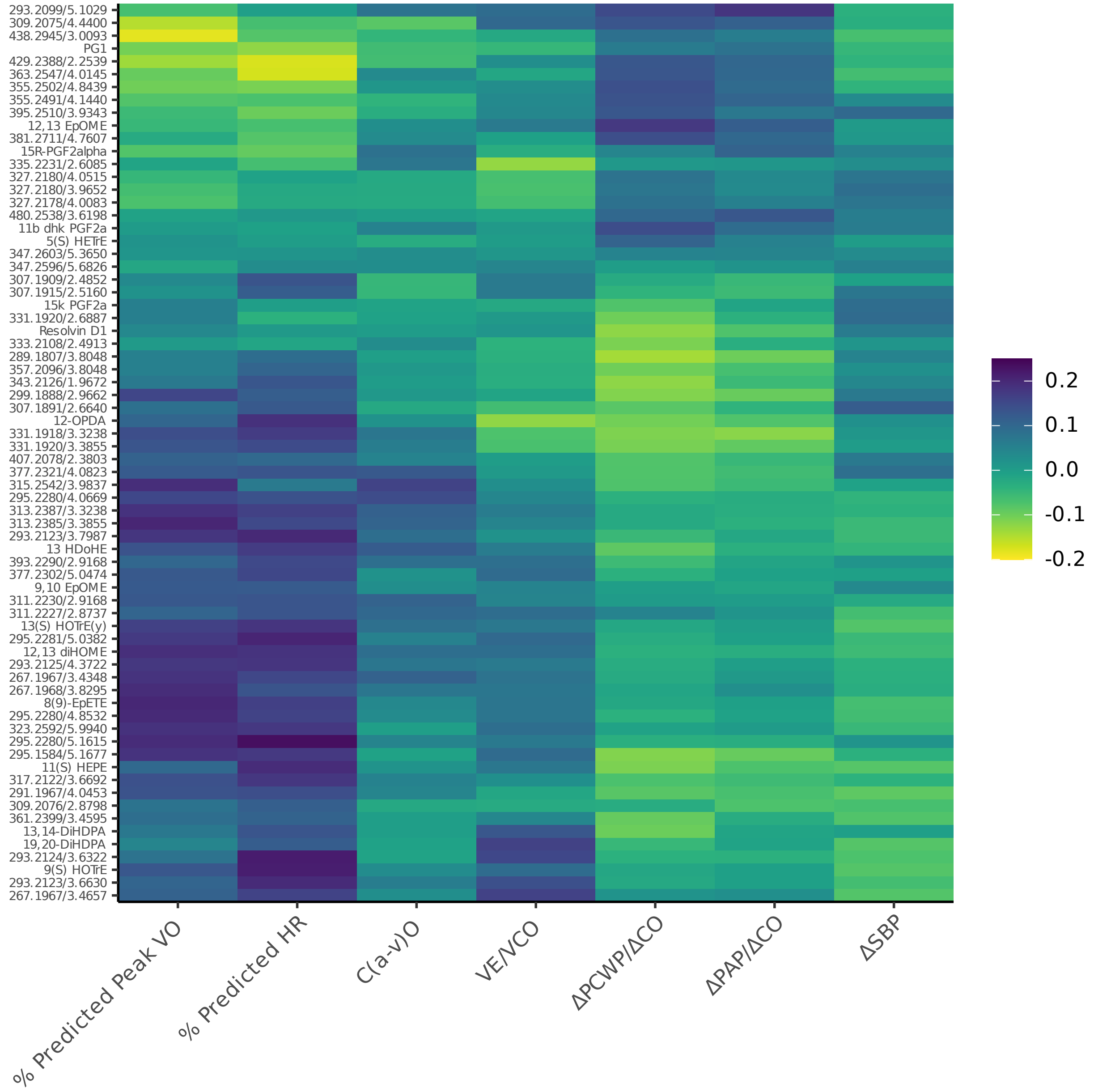

Supplemental Figure 2. Principal component analysis (PCA) examining the association of 890 eicosanoid and eicosanoid-related metabolites with 7 distinct exercise traits. PC scores for the first two PC components for each of the 7 exercise traits are displayed. Abbreviations: CO = cardiac output, HFpEF = heart failure with preserved ejection fraction, HR = heart rate, PAP = pulmonary artery pressure, PC = principal component, PCWP = pulmonary capillary wedge pressure, SBP = systolic blood pressure, VE/VCO_2 = ventilatory efficiency, VO_2 = oxygen uptake.

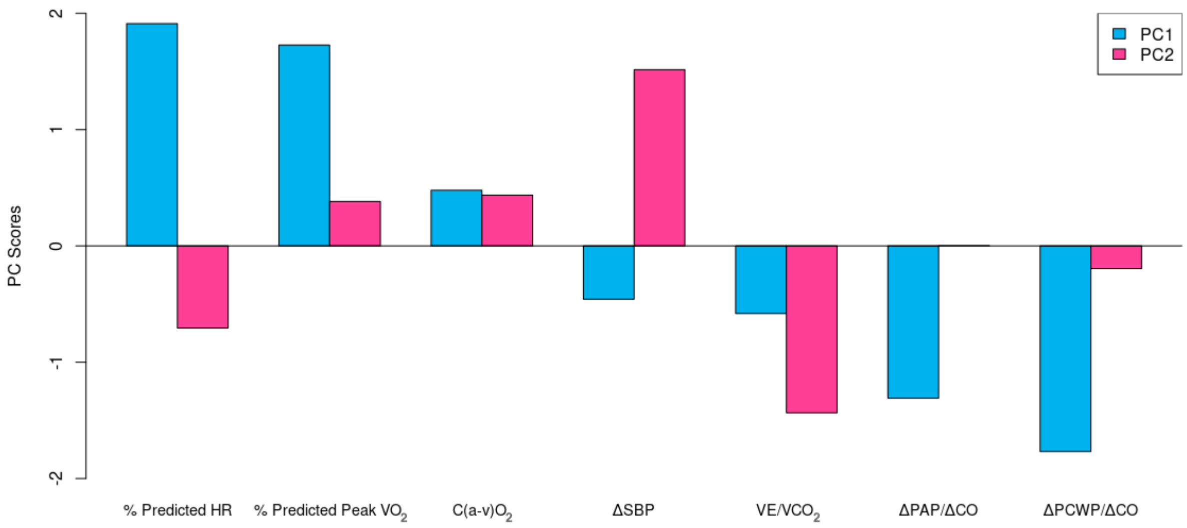

Supplement: Supplementary file 1 — Supplementary Information [file 41467_2023_43363_MOESM1_ESM.pdf]
