# Supplementary material for: Eicosanoid and eicosanoid-related inflammatory mediators and exercise intolerance in heart failure with preserved ejection fraction
Source: Nat Commun. 2023 Nov 20;14:7557. doi: 10.1038/s41467-023-43363-3 (PMC10662264; doi:10.1038/s41467-023-43363-3)
Supplement: Supplementary file 3 — Description of Additional Supplementary Files [file 41467_2023_43363_MOESM3_ESM.pdf]

Description of Additional Supplementary Files

Supplementary Data1. Baseline Clinical Characteristics in Participants in the MGH CPET and MESA Cohorts

Supplementary Data 2. Baseline Clinical Characteristics in Men vs Women in the MGH CPET Cohort

Supplementary Data 3. Baseline Clinical Characteristics in Men vs Women in the MESA Cohort

Supplementary Data 4. Eicosanoids Significantly Associated with HFpEF status (full list)

Supplementary Data 5. Association of Eicosanoids with Exercise Traits

Supplementary Data 6. Mediation Analyses (full list)

Supplementary Data 7. Incident HF Analyses from MESA (full list)

Supplementary Data 8. Incident HF Subtype Analyses from MESA (full list)
